# Wild Animal Ethics: The Moral and Political Problem of Wild Animal Suffering

**DOI:** 10.1017/awf.2023.12

**Published:** 2023-02-23

**Authors:** Elizabeth Mullineaux

**Affiliations:** RCVS Recognised Specialist in Wildlife Medicine (Mammalian) Wild Animal Welfare Committee

This is a slim volume of just 100 printed pages, but it delivers a big punch. The author does not shy away from the notion that this is a very personal assessment of wild animal suffering (WAS) and one that not everyone will agree with. His arguments however are well considered, referenced and, above all, written in a style that makes them accessible to all readers irrespective of their background in either philosophy, ethics, or animal welfare.

The basic premise of this book is that many animal species, especially those that reproduce with large litters with high mortality rates (r-strategists), have short lives in which they fail to flourish and die in often protracted and painful ways. The natural world is not a good place for most animals and most human interventions to reduce WAS focus on the larger iconic predator species (K-strategists). The author’s argument is that the scale of r-strategist suffering is such that humans have a moral duty to intervene. He argues against large-scale ecological destruction to reduce WAS and instead reasons that the preferred major intervention should be genetic modification, utilising CRISPR gene editing, to change dietary and reproductive strategies in both r- and K- strategists. It is a relief that the author acknowledges that “*significant research and testing would need to be conducted before beneficent gene drives could be conducted responsibly”*, recognising the potential for significant ecological damage – and potentially increased WAS. The positive arguments for gene editing are well made, but many readers may feel that human interventions would be better focused on reducing both direct and indirect anthropogenic impacts on WAS, which are considerable, rather than by ‘playing God.’ The author’s counter-argument is that such interventions are not enough and are too limited in vision to significantly reduce WAS.

There is much in this book that anyone interested in WAS will agree with, alongside ideas that many readers will find thought-provoking and potentially challenging. Ultimately, engaging with the subject matter and considering the arguments can only help develop a better understanding of the ways to alleviate WAS and that alone is reason enough to thoroughly recommend this text.

